# Evidence for “Whole Family Approach” in accelerating uptake of COVID-19 and routine immunizations among integrated primary health services in Nigeria

**DOI:** 10.3389/frhs.2023.1157377

**Published:** 2023-05-18

**Authors:** Chika Offor, Olympus Ade-Banjo, Chika Nwankwo, Grace Nwaononiwu, Faith Adukwu, Bibianna Egharevba, Joshua Owoyemi, Chibuike Odo, Marvellous Olatunji

**Affiliations:** ^1^ Vaccine Network for Disease Control, Abuja, Nigeria; ^2^ Garki Hospital Abuja, Abuja, Nigeria; ^3^ Nigeria Governors’ Forum, Abuja, Nigeria

**Keywords:** Whole Family Approach, family-centred health promotion, covid-19 vaccination in Nigeria, routine immunization in Nigeria, integrated primary health services

## Abstract

The family is the simplest unit but possesses the strongest bond in society. These qualities — bond and proximity — that exist both within and across neighboring families, according to our research, can be instrumental in shaping a new kind of health promotion strategy that can transform health behaviors in communities. The Whole Family Approach (WFA) is a government-sanctioned approach to increase uptake of COVID-19 vaccines in Nigeria. The approach entails leveraging the high family-based demand for some primary health services, such as malaria, diabetes, hypertension, and reproductive services, to generate demand for COVID-19 and routine immunizations. However, since the announcement in 2021, there has been no available evidence to show the impact of the approach on COVID-19 vaccine uptake, though global literature generally favors family-centered health approaches. This study tests the effectiveness of the approach in increasing the utilization of target services in a Nigerian community and further provides a theoretical framework for the strategy. Two primary healthcare facilities were selected in two communities located in Abuja in a quasi-experimental design. After a small-sample landscape assessment of the communities and the facilities, family-targeting health promotion activities were facilitated in the intervention community (integrated health education by trained community health influencers) and facility (opportunistic health promotion through in-facility referrals) for one month. Anonymized service utilization data were acquired from both facilities over a period of four months to analyze their respective month-by-month service utilization trends. Time trend analysis was conducted and revealed that WFA significantly increased service utilization (*N* = 5870; *p* < 0.001, *α *= 0.01, 99% CI) across all the package services provided at the intervention facility. A supplementary Pearson's correlation analysis further presented a positive relationship (*r* = 0.432–0.996) among the services which favored the result. It can therefore be concluded that the “Whole Family Approach” of health promotion is efficacious in accelerating uptake of priority health services such as COVID-19 and routine immunizations. While there is more to be understood about this interesting approach, we recommend the improvement of communication and capacity gaps in Nigeria's primary healthcare system to ensure that promising strategies such as the WFA are adequately implemented at the community and facility levels.

## 1. Introduction

Nigeria's Ministry of Health, through the National Primary Health Care Development Agency, announced the adoption of the Whole Family Approach as a measure to increase uptake of the COVID-19 vaccine (1). The agency mentioned that it was to retain focus on the holistic health of individuals and their families while looking to improve the uptake of the vaccines in the second phase after a challenging first phase characterized by low uptake, even among health workers (2). The scope of the WFA was to integrate COVID-19 vaccination into primary health services, such as childhood routine immunization, hypertension, diabetes, malaria, reproductive health, and malnutrition, so that when people visited primary health care facilities, primarily for any of these services, they and their eligible family members could also receive their COVID-19 vaccines.

This family-centered care is an approach in healthcare delivery in which the services are planned around the family rather than an individual (3). The approach has existed for many decades but is mostly dominant in pediatric care, where it originated (4). The idea solidified after the realization that parents can equally contribute to medical decision-making over their children. Equally, the Institute for Patient- and Family-Centered Care defined the approach as a partnership between the health service providers, patients, and their families. In all the existing definitions, family-centered care is observed to be conceptualized around decision-making on treatment and patients' information management. In the Whole Family Approach, however, family-centered care manifests primarily in the domain of health promotion.

While existing literature affirms that family-oriented health promotion and disease prevention strategies improved treatment outcomes in patients (5), reduced clinical workload, and increased staff satisfaction (6), it is not yet understood whether the Whole Family Approach or Family-Centered Care could improve the uptake of health services within the Nigerian primary healthcare system context. It is important to note that Nigeria's primary healthcare system is mostly positioned to serve rural and semi-urban communities (7), which are occupied by the majority (64%) of Nigeria's population (8), of whom 96% access healthcare through out-of-pocket health spending (9). Thus, this research is poised to explore the potential of the Whole Family Approach in health promotion to improve health-seeking behavior in a Nigerian community setting. Following the demand and supply model of health systems, the Whole Family Approach is conceptualized to increase health-seeking behavior while optimizing the healthcare delivery system to provide family-centered care. Thus, the implementation strategy employed in this study involves the use of family-targeting health messages while working with health facilities to create a family-centered environment.

### 1.1. Theoretical bases of the implementation research

The Whole Family Approach aligns with four different theoretical models and emphasizes the attempt to increase uptake of priority health services by simultaneously increasing key identified aspects of the factors of demand and supply. The models are the health-promoting family model (10), the Donabedian model (11), the health belief system (12), and Anderson's behavioral model for health services utilization (13). The health-promoting family model suggests that the family itself plays a critical role in the health promotion of its members. It suggests a new emphasis on the family's eco-cultural pathway (a range of activities that the family engages in which may affect the health of each family member), family health practices, and the family as actors (14). The Donabedian model underpins a method for the measurement of improvement in quality healthcare. The model is made up of four components: structure measures (these show the qualities of the staff/service to patient ratios and service hours), process measures (these show the way the structures and systems cooperate to deliver the intended outcomes), outcome measures (which measure the end result of quality care and if it achieved the aim it was set for), and balancing measures (these show the management of unforeseen or unintended positive or negative consequences and mitigates their impact if necessary) (15). The health belief model posits that the probability of individual adoption of a health behavior depends on the threat perception (susceptibility to and severity of a disease) and behavioral evaluation (concerning the efficacy and cost of adopting the health behavior) of the individual. Also, beyond the individual perception, individuals may need to be cued into successful adoption of a health behavior (12). The Anderson and Newman model for utilization of health services posits that the uptake of health services is a function of predisposing, enabling, and need factors. Need factors are a more immediate cause of healthcare service uptake and reflect the recognized or assessed health status of the individual. Enabling factors, such as individual or family income and wealth, refer to the resources and arrangements needed to acquire health treatments. Predisposing factors are an individual's socio-cultural features before illness, and they include culture, health beliefs, and demographic characteristics (13). These models are crucial to and play important parts in the formation of the strategies employed in this research. The adaptation of these models is illustrated below.

### 1.2. Description of intervention and the theory of change

In terms of poor uptake of health services, whether that is COVID-19 vaccines or childhood routine immunization, in developing countries, the challenges have been simplified along the lines of demand and supply (16). The demand side describes the choice of individuals or groups to seek health services, while the supply issues relate to accessibility, availability, and quality of health service delivery. Factors of demand are within the control of the individual or unit of individuals. They include household geographical location, indirect cost of care, ability to pay for services, individual and community attitude and perception towards healthcare/cultural preferences and norms (1), and so on. Proximal factors are factors that directly affect the individual's choices, such as attitude towards healthcare and other preferences, while distal factors have intermediate effects and are further away from the individual, such as the individual's religious and traditional environment, social and economic status, gender power dynamics, and level of education (17).

Supply-related factors are service delivery factors that ultimately impact whether the clients can access, utilize, and continue the uptake of health services by the health system. They include the location of the healthcare service, attitude of the healthcare workers, staff management and effectiveness, direct cost of services, availability of drugs and related items, and functionality of payment systems (1). More frequently than not, the factors of demand and supply are dependent on each other and thus come together to influence the rate of uptake of health services.

Therefore, in this study, the Whole Family Approach is deployed in the demand and supply components of family and community health. For the demand side, trackable Information Education and Communication (IEC) print materials are deployed through designated health announcers in each community and facility. On the supply end, facilities are primed with training and data collection tools to provide and document health services.

This approach aims to close the gap between people and health services through integrated health promotion in the communities and opportunistic health promotion in the facilities [see theory of change]. Integrated health promotion programs combine two or more topics within health education or demand generation protocol and have been proven to improve health behavior (18). In this case, the services are outlined on a flier bearing a picture of parents and a child to depict the family-centeredness of the program at first glance. This aspect of health promotion is carried out by trained community health influencers who educate households on the benefits of the whole-family health service package at the primary health center located within their community.

**Figure 1:**
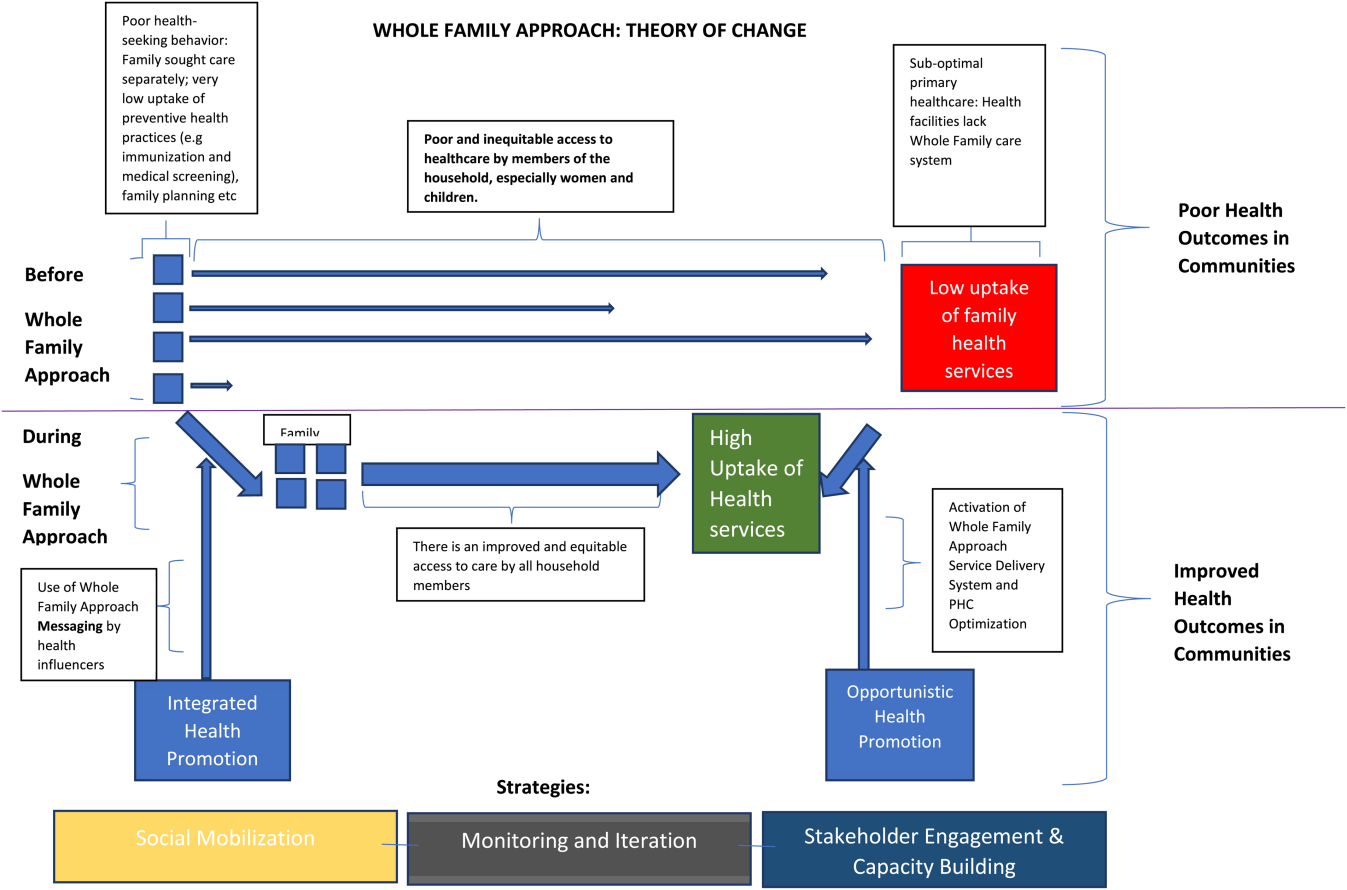
Theory of change for the whole family approach.

The other strategic domain for health promotion is within the health facility, known as opportunistic health promotion. This form of health promotion is supported by a number of empirical studies in clinical setting (19, 20). Here, health service providers are primed to refer families of patients to services other than their sought-after health service. For instance, a parent who brings his or her child for routine immunization may be advised by the child immunization officer to consider taking their COVID-19 vaccine or receive counselling on family planning. Printed banners are also placed at conspicuous points in the facilities to opportunistically prompt health demand for the key services in the package. The alternative before Whole Family Approach illustrates theoretically poorer access to primary healthcare in an individualized approach to primary healthcare, leading to poor health outcomes in communities (see Figure 1).

The service package in this implementation research comprised reproductive health services, malaria, hypertension, diabetes, childhood routine immunization, and COVID-19 vaccination. However, the approach itself can be employed to support a diverse health service package, including in an epidemic response setting such as COVID-19.

## 2. Method

### 2.1. Study design

This was a quantitative study based on a quasi-experimental design. Specifically, this design is defined by Miller et al. as pre-post with non-equivalent control group style (21). In this study, two facilities—one intervention, one control—with relative similarities are purposively selected for the study. This design is selected to compare mean differences using a time series analysis within and between the two facilities.

### 2.2. Study setting

**Figure 2:**
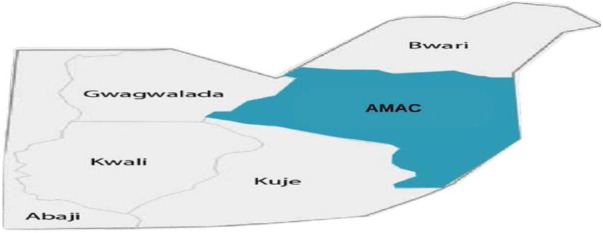
Geographical mapping of federal capital territory.

The Federal Capital Territory (FCT), also known as Abuja, is the administrative capital of Nigeria. Located in the north-central geopolitical zone of Nigeria, it comprises six area councils and 62 (22) political wards. Abuja is inhabited by an estimate of 3,652,029 (23) people, of which the majority reside in its municipal area. The Abuja Municipal Council Area (AMAC) represents more than half of the FCT's population, while the rest is shared among the five other area councils: Kuje, Kwali, Bwari, Gwagwalada, and Abaji (see Figure 2). This research is geographically scoped within the municipal area as it provides a cross-sectional collection of most demographics (24, 25) not only in the FCT but in Nigeria. The key health services considered in this research are COVID-19 vaccination, routine immunization, nutrition, malaria, reproductive health services, and Non-Communicable Disease (NCD) screening services, especially Diabetes and Hypertension.

### 2.3. Study participants

The most crucial aspect of the study used the summary databases of the intervention and control facilities to measure the utilization trend in the two facilities. In this process, the databases accounted for at least 8,339 participants—5,870 participants in the intervention facility, and 2,469 in the control facility. The research had no access to further demographic information on the participants who took up these services to avoid breach of patient data protection. In the small-sample landscape assessment, the non-probability sampling did not require calculating minimum sample size. For the study, 20 adult participants -10 female community members, six male community members, three female facility managers, and one male facility manager—were recruited to answer a survey on the knowledge, attitude, and practice of the Whole Family Approach in the intervention facility's community. Only community members who were adults (18 years and above) and resided in the community at the time of the study were eligible to participate in the survey.

### 2.4. Recruitment of community health influencers

Two community influencers (one male, one female) attached to the intervention facility were trained and recruited to carry out the awareness creation for this approach in the community.

### 2.5. Data collection

The data collection for this study spanned over a period of four months (May - August) in 2022. Data were collected in two stages: the landscape assessment and facility utilization data.

#### 2.5.1. Landscape assessment

The landscape assessment was conducted in the intervention community for two days. Specifically, we were interested in understanding whether the participants were aware of the government's WFA program and if they were interested after a brief explanation of the approach by data collectors. Given that Hausa speakers predominate in the research areas, the surveys were written in English and facilitated in the local tongue for improved comprehension. Trained data collectors from the research team were responsible for collecting the data from each participant. Data were collected simultaneously in prepared google forms and paper questionnaires. The small-sample assessments were conducted to obtain a basic understanding of the level of awareness about the WFA as well as the readiness of the facility to provide the services. It provided context to the intervention research without forming the evidence basis for the impact of WFA in increasing uptake of primary health services.

#### 2.5.2. Service utilization data

The end-line stage of the data collection involved collection of service utilization data from the facilities over a period of 4 months, inclusive of three months pre-intervention and one month of intervention. Routine data from the facility register was used to capture service utilization data for this study instead of introducing an alternative data instrument at the facilities. The facility summary data provide utilization information of the facility without disclosing the private medical information of the patients. Kane et al (26). supported the use of routine facility data for most studies carried out within the clinical setting.

### 2.6. Data analysis

Following the validating of the data by comparing the paper-documented data with the electronically computed data, the landscape assessment and the service utilization data were analyzed using a set of data analysis tools, namely Microsoft Excel and IBM's Statistical Package for Social Sciences (SPSS). The landscape assessment data were represented in simple descriptive statistics while the service utilization data were processed through a few steps of data analysis to extract detailed understandings. The first analysis was to examine if there was a significant impact on the trend of service utilization in the intervention facility compared to the control facility. So, a chi-squared test was used to analyze a comparative time trend between data from intervention and control facilities. The data input across the four months of both facilities were converted to percentage. Therefore, the percentile distribution in the control facility was used to model the expected rates in the intervention dataset. The second step of analysis was to confirm that the first result was due only to the month of intervention (Month 4 or M4). To achieve this, pre-intervention percentile distribution in the control facility was used to model the expected rates of distribution in the intervention dataset. The third step of analysis was a Pearson's correlation analysis run across the services to test the relationship between the services.

### 2.7. Ethical consideration

The project team obtained ethical approval from the Research Ethics Committee of the Health and Human Services Secretariat (HHSS) of the Federal Capital Territory in Nigeria. During data collection for landscape assessment, oral consent was obtained from each participant using a standard verbal consent script that highlighted identity and confidentiality protection. Oral consent was also obtained in local languages where necessary.

## 3. Results

### 3.1. Landscape assessment

For the interviews, 16 randomly selected respondents were chosen. Families who had at least one of the criteria were included: they were married, had a pregnant woman, or had an child under the age of 5 in their household. The questions assessed the respondents' knowledge of and attitude to the whole family approach in the community. All participants were aware of the location of primary health care centers in their communities and, upon further inquiry, used them for basic health care consultation and treatment. The majority of the respondents were unaware of the whole-family approach. 87.5% were aware of an integrated family approach, which entails individuals going along with their families to health centers for joint family care, but not specifically of the government's initiative to do so. The idea was widely accepted, with 81.3% of participants expressing a willingness to be a part of the integrated family approach if the opportunity arose. The rest expressed reluctance to participate in the idea, perhaps due to perceived barriers such as cost of the services, time availability, and documentation fatigue at primary health care centers (see Table 1).

**Table 1:** Baseline assessment for level of knowledge and facility readiness.

Question	Yes % (*n*)	No % (*n*)
Community		
Men	37.5 (6)	62.4
Women	62.5 (10)	37.5
Do you know the PHC in your community?	100 (16)	0 (0)
Do you know about the WFA?	12.5 (2)	87.5 (14)
Would you be interested in the WFA?	81.3% (13)	17.7 (3)
Facility		
Men	25 (1)	
Women	75 (3)	
Do you know about the WFA?	25 (1)	75 (3)
Does your facility provide WFA package services?	100 (4)	0 (0)

The baseline facility assessment on the practice of WFA showed that three out of four managers were unaware of the whole-family approach. However, the two facilities were fully equipped and provided basic health care services (see Table 2).

**Table 2:** Month-by-month service utilization rates in intervention and control facilities.

	Intervention (GOSA)					Control (DAMAGAZA)				
Services	M1	M2	M3	M4	Total	M1	M2	M3	M4	Total
Covid-19	19	12	10	72	113	8	4	7	8	27
% Distribution	17	11	9	64		30	15	26	30	
% Change	-	−6	−2	55		-	−15	11	4	
RH	106	123	112	355	696	61	46	70	48	225
% Distribution	15	21	16	51		27	20	31	21	
% Change	-	4.8	−5	35		-	−7	9	−10	
Malaria	22	31	26	103	182	105	159	83	125	472
% Distribution	13	17	14	57		22	34	18	26	
% Change	-	4	−3	43		-	12	−16	8	
Diabetes	5	3	6	13	27	11	7	5	9	32
% Distribution	19	11	22	48		34	22	16	28	
% Change	-	−8	11	26		-	−12	−6	12	
Hypertension	6	6	6	16	34	88	90	51	98	327
% Distribution	18	18	18	47		27	28	16	30	
% Change	-	0	0	29		-	1	−12	14	
RI & Nutri	1,161	1,219	1,151	1,287	4,818	295	328	399	364	1,386
% Distribution	24	25	24	27		21	24	29	26	
% Change	-	1	−1	3		-	3	5	−3	
All Services	1,319	1,394	1,311	1,846	5870	568	634	615	652	2,469
	22.47	23.75	22.33	31.45		23.01	25.67	24.91	26.4	
	-	1.28	−1.42	9.12		-	2.66	−0.76	1.49	

M, Month; RH, Reproductive Health; RI, Routine Immunization; Nutri, Nutrition. Table 2 shows that the highest uptake of services across months was recorded in the month of the intervention (M4). Also, COVID-19 vaccine uptake increased by 55% against previous months where uptake declined by 2% and 6% respectively. The increase in percentages of 35%, 43%, 26%, 29%, and 3% were similarly observed for reproductive health, malaria, diabetes, hypertension and routine immunization services, respectively. [Note: Routine immunization and nutrition (vitamin A administration) are recorded combined in the facility Summary Data in Nigeria's public primary health facilities].

### 3.2. Comparison of service utilization between intervention and control facilities

**Figure 3:**
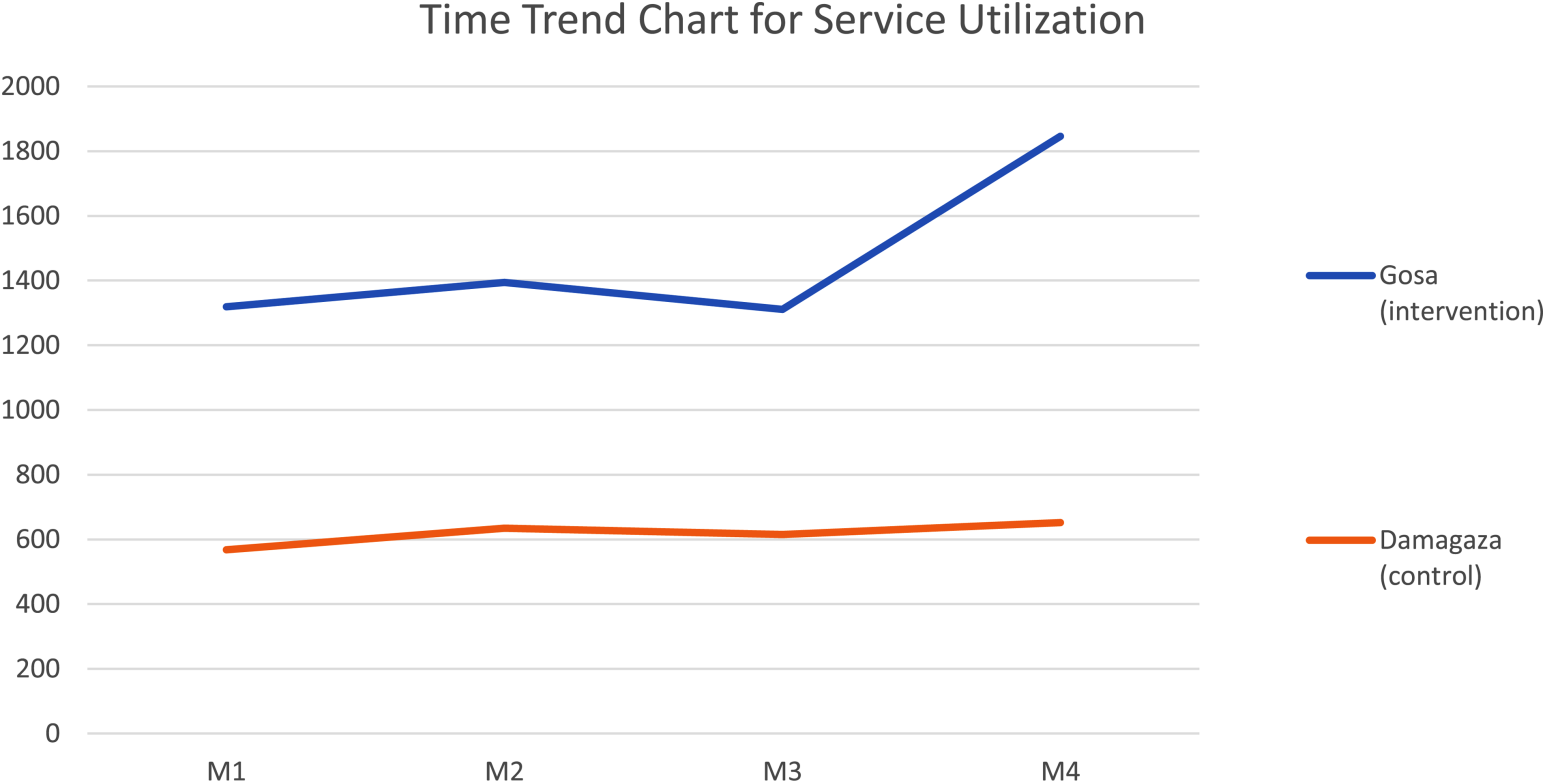
Time trend chart for month-by-month service utilization rates in intervention and control facilities. There is a significant increase (9.12%) in the trend of utilization of selected services facility (*N* = 5,870; *p* < 0.001, *α *= 0.01, 99% CI).

Month-by-month summary data was obtained from the intervention and control facilities (see Table 2) and was then subjected to a descriptive analysis presented in Figure 3. Not that, because nutrition services are integrated with routine immunization services to children in facilities, the summary data obtained from the facilities yielded combined uptake rates for both services. The aim of collecting service utilization data is to determine if there will be a significant increase in the uptake of the WFA services in Month 4 at the intervention facility. To achieve this, therefore, percentile distribution of the month-by-month rates of total service uptake was analyzed for each facility. The control facility recorded 23.01%, 25.67%, 24.91% and 24.40%, while intervention facility recorded 22.47%, 23.75%, 22.33% and 31.45%. With the exception of the Month 4 ratio at the intervention facility (also known as Intervention M4 ratio), there is an observed evenness of +/-2% relative difference in successive ratios across the intervention and control facilities, showing an appreciable level of similarity enough to model one facility after the other and run a chi-square test. Although there is a seemingly significant difference (9.12%) between intervention M4 ratio when compared to its preceding month ratio (22.33%), chi-square statistics helped to determine the statistical level of significance of this difference (*p* <0.001, *α*=0.01) at 99% confidence interval (CI).

### 3.3. Relationship within the WFA services package

The third level of analysis in this study is testing the level of relationship within the services in the WFA package deployed in the intervention facility. The Pearson's correlation analysis of individual service ratios is presented in Table 3, indicates that, although there is generally a positive relationship among the services (*r* = .452 - .949**), COVID-19, an adult (age 18 and above) health service in Nigeria, significantly correlated other services related to adults namely reproductive health (*r*=.972*), malaria (*r*=.851), diabetes (*r*=.952**), and hypertension (.961*), but not significantly with routine immunization and nutrition (*r*=.432) which are exclusively provided to children (0-5 year old).

**Table 3:** Pearson's correlation analysis among the chosen services for the whole family approach testing.

	Covid-19	RH	Malaria	Diabetes	Hypertension	RI
Covid-19	1					
RH	.972*	1				
Malaria	.851	.949**	1			
Diabetes	.952**	.964*	.905**	1		
Hypertension	.961*	.996*	.962*	.974*	1	
RI	.432	.540	.707	.581	.611	1

There is a positive (+) correlation across all the services.

* Correlation is significant at the 0.01 level (2-tailed).

** Correlation is significant at the 0.05 level (2-tailed).

## 4. Discussion

A small-sample landscape assessment informed the project team of the low level of awareness of the community members about the government-sanctioned approach. Most of the facility managers also had limited knowledge about the program despite offering the services required to implement the approach. The project team identified a communication gap between the facility managers and the agency leaders which motivated the team to organize a capacity-building program for all the facility managers with the attendance of the agency leaders to emphasize the need to strengthen top-bottom communications in the primary health system. The project team further visited the facility to train staff about the approach, especially the community health influencers. A 6 ft-by-5 ft flex-banner was erected at the entrance of the facility to facilitate in-facility referrals for the services.

At the end of the 1-month community sensitization using the 100 tracked fliers distributed by the community influencers, trend analysis from the 4-month facility utilization data harvested from the facility summary registers revealed that the Whole Family Approach significantly increased total uptake of services in the local primary health facility during the month of intervention. Similar results had been obtained using WFA to improve the weight profiles of children in a clinical study carried out in the United Kingdom (27). Health promotion activities are generally expected to increase health service uptake whether in lowering blood pressure (28) or in improving health outcomes in adults with developmental disabilities (29). Some studies measured the impact of health promotion and education activities through behavioral changes before and after the intervention (30) while others measured impact through service utilization or both (31). Since health promotion activities in Nigeria are rarely measured and published with empirical data, it is difficult to compare this program's outcomes to other related programs.

The program was implemented on a small scale, influenced by limited availability of resources and administrative compliance. However, it manages to present evidence for the efficacy of the approach as well as establish a positive association in the package services selected for the study. The efficacy of the approach was ascertained through the pre-post non-equivalent control group design (Figure 3) while a Pearson's correlation analysis affirms that the incorporated services in this program were suitable for family-targeted health promotion (Table 3).

Although there is no significant level of correlation between the rates of uptake of COVID-19 and routine immunizations due to WFA, the positive association observed makes a moderate case for the integration of routine immunization and COVID-19 vaccination as Nigeria is faced with low rates of uptake of both services (32, 33).In addition, Nigeria in 2021 had an estimated 3.1 million (∼14%) (34) zero-dose children, which may have been exacerbated by the COVID-19 pandemic (35), as well as other challenges such as insecurity (36)—both of which have disrupted health services in many affected areas. However, in the wake of efforts to integrate services to increase uptake of these essential immunization programs, the positive relationship in the uptake of these services among other primary health services occasioned by WFA can be effectively leveraged.

## 5. Limitations

The formative study was limited by funds, thereby limiting the number of facilities and communities included to test the approach. Beyond that, some implementation challenges were experienced in the team's objective to track the use of IEC material with the utilization of the services. Tracking the IEC material with details of households reached helped to ensure that the contracted community health influencers actually achieved the target number of households. However, the project team could not fully measure how much of the increase in uptake can be attributed directly to either the community sensitization or the opportunistic health promotion within the facility.

Also, data collection for the service utilization data was limited to the facility summary data register which lacked demographic details such as sex- and age-disaggregated data.

## 6. Conclusion

The Whole Family Approach of integrated health promotion generated a significant increase in the utilization of six family-targeting services in a suburban community at the heart of Nigeria's federal capital territory. This study should inform intermediate adoption and expansion of the strategy based on the stated evidence and implementation guidelines. With respect to future need for the strategy, it is important to state that the whole-family approach is capable of a wide range of flexibility in the mix of services, however, standards for choosing a services package is yet to be established. Conclusively, it would be fulfilling to see future studies done to strengthen the developed framework and implementation steps, and also obtain results across a wider variety of facilities and communities with stronger arrangement with facilities to obtain demographic information without breaching patients' medical privacy.

## Data Availability

The original contributions presented in the study are included in the article, further inquiries can be directed to the corresponding author.
